# Engineering Biomimetic Nanoparticles through Extracellular Vesicle Coating in Cancer Tissue Models

**DOI:** 10.3390/nano13243097

**Published:** 2023-12-07

**Authors:** Gema Quiñonero, Juan Gallo, Alex Carrasco, Josep Samitier, Aranzazu Villasante

**Affiliations:** 1 Institute for Bioengineering of Catalonia (IBEC), The Barcelona Institute of Science and Technology (BIST), 08028 Barcelona, Spain; 2 Advanced Magnetic Theranostic Nanostructures Laboratory, International Iberian Nanotechnology Laboratory (INL), 4715-330 Braga, Portugal; 3 Department of Electronic and Biomedical Engineering, University of Barcelona, 08028 Barcelona, Spain; 4 Biomedical Research Networking Center in Bioengineering, Biomaterials, and Nanomedicine (CIBER-BBN), 28029 Madrid, Spain

**Keywords:** neuroblastoma, extracellular vesicles, iron oxide nanoparticles, biomimetic models, precision medicine

## Abstract

Using nanoparticles (NPs) in drug delivery has exhibited promising therapeutic potential in various cancer types. Nevertheless, several challenges must be addressed, including the formation of the protein corona, reduced targeting efficiency and specificity, potential immune responses, and issues related to NP penetration and distribution within 3-dimensional tissues. To tackle these challenges, we have successfully integrated iron oxide nanoparticles into neuroblastoma-derived extracellular vesicles (EVs) using the parental labeling method. We first developed a tissue-engineered (TE) neuroblastoma model, confirming the viability and proliferation of neuroblastoma cells for at least 12 days, supporting its utility for EV isolation. Importantly, EVs from long-term cultures exhibited no differences compared to short-term cultures. Concurrently, we designed Rhodamine (Rh) and Polyacrylic acid (PAA)-functionalized magnetite nanoparticles (Fe_3_O_4_@PAA-Rh) with high crystallinity, purity, and superparamagnetic properties (average size: 9.2 ± 2.5 nm). We then investigated the internalization of Fe_3_O_4_@PAA-Rh nanoparticles within neuroblastoma cells within the TE model. Maximum accumulation was observed overnight while ensuring robust cell viability. However, nanoparticle internalization was low. Taking advantage of the enhanced glucose metabolism exhibited by cancer cells, glucose (Glc)-functionalized nanoparticles (Fe_3_O_4_@PAA-Rh-Glc) were synthesized, showing superior cell uptake within the 3D model without inducing toxicity. These glucose-modified nanoparticles were selected for parental labeling of the TE models, showing effective NP encapsulation into EVs. Our research introduces innovative approaches to advance NP delivery, by partially addressing the challenges associated with 3D systems, optimizing internalization, and enhancing NP stability and specificity through EV-based carriers. Also, our findings hold the promise of more precise and effective cancer therapies while minimizing potential side effects.

## 1. Introduction

Neuroblastoma (NB) is a rare malignant tumor arising from neural crest cells, which are the precursors of the sympathetic nervous system. Typically diagnosed in early childhood, it predominantly originates in the adrenal medulla but can occur anywhere within the sympathetic nervous system, primarily in the abdominal region. Despite advances in surgical interventions and chemotherapy, the urgent need for more effective therapeutic approaches persists [1].

The use of nanoparticles (NPs) for drug delivery has shown promising therapeutic potential in various cancer types [2,3]. However, the effectiveness of NP-based therapies faces significant hurdles. Modifications of NPs with biomolecules, particularly antibodies, to enhance cancer cell targeting are hindered by the spontaneous formation of a protein corona in the bloodstream. This phenomenon affects NP behavior and compromises their targeting ability over time [4]. Moreover, the introduction of targeting ligands may trigger an immune response, leading to increased immune-mediated clearance of NPs from the bloodstream and tissues [5]. Additionally, relying solely on a single specific antibody or targeting ligand may prove ineffective due to the phenotypic diversity of tumors [6].

In response to these challenges, an innovative approach has emerged—biomimetic nanoparticles [7,8]. These biomimetic NPs offer a unique combination of desirable traits, including biocompatibility; immune evasion inspired by natural materials such as toxins, pathogens, cells, or cell membranes; and the customizable properties of synthetic nanoparticles. Cell membranes, specifically those sourced from entities like red blood cells, cancer cells, platelets, or white blood cells, have been utilized to coat NPs [9,10]. However, a recent and promising trend involves using extracellular vesicles (EVs), including exosomes, as the natural material for biomimetic NP preparation [7,8].

EVs, small membrane vesicles secreted by all cell types, play a pivotal role in intercellular communication [11]. They come equipped with surface membrane ligands that efficiently bind to specific receptors on recipient cells, ensuring precise cellular uptake and high targeting specificity. The nature of these surface ligands varies depending on the source of the EVs [11]. Given their intrinsic targeting capabilities and low immunogenicity, EVs have emerged as an ideal surface coating for obtaining biomimetic NPs [7,8,9,10,11,12]. Several techniques for encapsulating NPs within EVs have been documented, including passive incubation, freeze/thaw cycling, surface conjugation, extrusion, electroporation, and sonication [12,13]. An emerging approach involves labeling parent cells with NPs, facilitating the generation of EV-coated NPs. This method entails the direct incubation of parent cells with NPs, followed by cell internalization, integration of NPs into the EVs’ biogenesis pathway, and subsequent release of NP-loaded EVs into the culture medium. Nevertheless, evaluating the effectiveness of this approach remains a challenge, with limited studies [12].

The source of EVs plays a crucial role in achieving precise and effective tumor targeting for therapy. NPs coated with EVs must replicate the natural surface ligands of EVs obtained from patients to achieve accurate targeting. Presently, tumor-derived EVs are primarily isolated from monolayer cell cultures. However, recent research underscores the significance of factors such as 3D context, composition, and the stiffness of the cellular environment in regulating the size and content of EVs, particularly their surface ligands [14]. These findings emphasize the necessity of obtaining EVs from cells cultured within a native-like environment, rather than simplistic cell monolayers, to optimize the potential for successful tumor targeting.

To address this gap, we propose a novel strategy using tissue-engineered models (TE-models) as the source for obtaining biomimetic EV-coated NPs, with a specific focus on neuroblastoma as the target cancer. In this study, we labeled NB cells cultured within a 3D TE-model context with metallic nanostructures. Among various metallic NPs, iron oxide nanoparticles (IONs) are particularly promising for biomedical applications [7,15]. They possess the unique capability of converting light into heat and exhibit magnetism, making them suitable as magnetic resonance imaging (MRI) contrast agents and magnetic hyperthermia effectors. These properties, combined with biocompatibility, biodegradability, low toxicity, and commercial availability, position magnetite NPs as excellent candidates for NP-loaded EV preparation [7,16].

## 2. Materials and Methods

Reagents. We used the following reagents for the synthesis, functionalization, and characterization of the nanoparticles, as well as in the preparation of tissue-engineered models and subsequent experiments: Ferrous chloride tetrahydrate (FeCl_2_·4H_2_O; Sigma–Aldrich Co., Ltd., Gillingham, UK); ferric chloride hexahydrate (FeCl_3_·6H_2_O; Sigma–Aldrich Co., Ltd., UK); poly(acrylic acid sodium salt) (PAANa; Sigma–Aldrich Co., Ltd., UK); ammonium hydroxide (NH_4_OH 28–30%; Sigma–Aldrich Co., Ltd., UK); sulforhodamine 101 cadaverine (Quimigen, Madrid, Spain); glucosamine hydrochloride (TCI); collagen I (Col I, Corning #354249, Corning, NY, USA); sodium hyaluronate (HA) within the 8–15 kDa range (Contipro# 600-01-01); N-Hydroxysuccinimide (NHS; Sigma–Aldrich Co., Ltd., UK); and N-(3-Dimethylaminopropyl)-N′-ethylcarbodiimide hydrochloride (EDC; Sigma–Aldrich Co., Ltd., UK)

Equipment. The following equipment was utilized for nanoparticle synthesis, functionalization, and characterization: Horiba Scientific Nanopartica SZ-100 (Kyoto, Japan) for dynamic light scattering (DLS), a JEOL JEM-2100 High-Resolution Transmission Electron Microscope (HR-TEM; Tokyo, Japan) for imaging, an X’Pert PRO diffractometer (PANalytical, Tokyo, Japan) for X-ray diffraction (XRD), an ICPE-9000 Multitype ICP Emission Spectrometer (Shimadzu, Kyoto, Japan) for ICP-OES, a Horiba Scientific Fluoromax-4 instrument for fluorescence spectroscopy, a Superconducting Quantum Interference Device (SQUID; Quantum Design, Toshima City, Japan) for magnetic properties characterization, and a thermogravimetric Analyzer STARe system (Mettler Toledo, Columbus, OH, USA) for Thermogravimetric Analysis (TGA). An Alpha 1–4 LD lyophilizer (Christ, Frankfurt am Main, Germany) was used to prepare the ColI/HA scaffold materials.

Nanoparticle synthesis. Polyacrylic acid-coated iron oxide magnetic nanoparticles (Fe_3_O_4_@PAA NPs) were synthesized in an aqueous medium following a modified hydrothermal protocol. Briefly, ferrous chloride tetrahydrate (1.59 g, 8 mmol) and ferric chloride hexahydrate (3.78 g, 14 mmol) were dissolved in milliQ water (10 mL). In parallel, PAANa (2.00 g, 0.4 mmol) was dissolved in 5 mL of milliQ water. Both solutions were thoroughly mixed, and ammonium hydroxide (15 mL) was added. The resulting black solution was transferred to a 40 mL poly(tetrafluoroethylene) (PTFE) vessel and heated to 150 °C for 48 h inside a stainless-steel autoclave reactor. After this time, the reaction was allowed to cool down to room temperature before the sample was transferred to (2×) 50 mL falcon tubes, the volume was completed to 45 mL with acetone, and it was centrifuged at 8000 rpm for 5 min. This process was repeated three times in total. Finally, the pellets were re-suspended in 15 mL of water each and were centrifuged for 5 min at low speed (3000 rpm) to remove large aggregates/particles. The pellet was discarded this time, and the supernatant was kept in the fridge until further use.

Nanoparticle functionalization. Some 1 mL of the previous sample was diluted with 4 mL of milliQ water. To this solution, a solution of sulforhodamine 101 cadaverine was added and thoroughly mixed. Then EDC was added. The reaction was allowed to proceed overnight in the dark at room temperature. Next day, the sample was transferred to Eppendorf tubes (0.5 mL each), precipitated with acetone (1.5 mL per tube), and separated via centrifugation (13,400 rpm, 5 min). The pellet was resuspended in milliQ water (0.2 mL). This process was repeated a total of three times. Finally, all the pellets were resuspended in milliQ water and combined together (total volume, 5 mL). To this solution, 50 mg of glucosamine hydrochloride (TCI) was added, followed by EDC (excess). The sampled was then stirred in the dark overnight at room T. To purify the sample, the same procedure described above was followed. Finally, the pellets were resuspended in milliQ water, combined, and stored in the fridge in the dark until further use.

Nanoparticle characterization. The nanoparticles obtained and functionalized as described above were fully characterized from a physico-chemical point of view.

Thermogravimetric data were acquired with a STARe system using concentrated water NP solutions. Hydrodynamic diameters and ζ-potential values of the nanoparticles were determined by dynamic light scattering (DLS) measurements. Transmission electron microscopy images (TEM) were obtained with a JEOL JEM-2100 microscope (JEOL Ltd., Tokyo, Japan) at an accelerating voltage of 200 kV. X-ray diffraction (XRD) data were collected with an X’Pert PRO diffractometer set at 45 kV and 40 mA, and equipped with CuKα radiation (λ = 1.541874 Å). XRD UV/Vis spectra were recorded by using a Shimadzu UV-2550 UV/Vis spectrophotometer. Fe concentrations were measured by inductively coupled plasma optical emission spectroscopy (ICP-OES) with an ICPE-9000 Multitype ICP Emission Spectrometer. Fluorescence spectra were recorded with a Horiba Scientific Fluoromax-4 instrument using quartz cuvettes (λexc = 450 nm, slits = 5 nm). Hysteresis loops in the applied magnetic field range from −20 to +20 kOe at room temperature, and field-cooled–zero-field-cooled data between 4 and 300 K at a constant 100 Oe field were measured with a superconducting quantum interference device. For the sample preparation, dried NPs (5 mg) were placed in gelatin capsules, introduced into standard straw sample holders, and attached to a measuring rod.

Scaffold preparation. Highly porous collagen-based scaffolds (ColI/HA) were synthesized using freeze-drying. The scaffold composition encompassed collagen I and sodium hyaluronate within the 8–15 kDa range. A solution of 1% HA (*wt*/*v*) was prepared in distilled water, following an established protocol. After lyophilization overnight at −50 °C, 0.1200 mbar, the scaffolds underwent a cross-linking process within 95% ethanol incorporating 33 mM EDC and 6 mM NHS for 4 h at 25 °C. Then, the scaffolds were washed in distilled water to eliminate residual reactants and frozen, followed by lyophilization overnight.

### 2.1. Cell Culture

Neuroblastoma cells. SK-N-BE(2) cells (European Collection of Authenticated Cell Cultures (ECACC; Cat.No. 95011815) were cultured in RPMI-1640 medium (Sigma, R8758) supplemented with 10% (*v*/*v*) FBS and 1% penicillin/streptomycin (RPMI expansion medium). Cells were cultured at 37 °C and 5% CO_2_ in a humidified incubator.

MTS assay. MTS cell proliferation assay in monolayer was performed using a CellTiter 96^®^ AQueous One Solution kit (Promega, G3582, Madison, WI, USA), following the manufacturer’s protocol.

**Figure 1:**
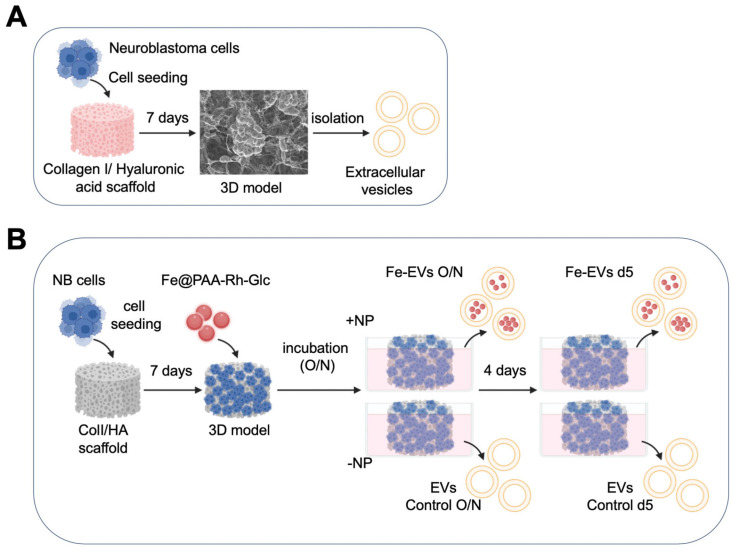
Methodology Scheme. (**A**) Schematic representation of the scaffold seeding process. Collagen I/hyaluronic acid (Col1/HA) scaffolds were sterilized, hydrated in RPMI-1640 medium, and seeded with 1 × 10^6^ neuroblastoma cells in exosome-free culture medium. These cell-seeded scaffolds were cultured for 7 days to establish a 3D neuroblastoma tissue-engineered model (3D model). Extracellular vesicles were subsequently isolated from the supernatant. (**B**) Overview of iron nanoparticle treatment and extracellular vesicle isolation. The 3D models on day 7 were exposed to iron nanoparticles either overnight (O/N) or for 5 days (day 12 after scaffold cell seeding). Following this treatment, supernatants were collected for extracellular vesicle (EV) isolation.

Scaffold seeding. Col1/HA scaffolds were seeded as previously reported. Briefly, biomaterials were sterilized in ethanol 70% for 1 h, dried with sterile tech wipes, and placed in RPMI RPMI-1640 (Sigma, St. Louis, MO, USA, R8758) for another hour. Then, 1 × 10^6^ neuroblastoma cells were seeded within the scaffolds in exosome-free culture medium containing RPMI-1640 medium supplemented with 10% (*v*/*v*) exosome-depleted FBS (Gibco, Waltham, MA, USA) and 1% penicillin/streptomycin. Cell seeding was performed at 37 °C and 5% CO_2_ for 4 h in a rotatory platform. Finally, cell-seeded scaffolds were transferred to non-treated 24-multiwell plates (ThermoFisher Scientific-Nunc, Rochester, NY, USA) and cultured in 2 mL of exosome-free culture medium at 37 °C/5% CO_2_ for 7 days to obtain a neuroblastoma tissue-engineered model (NB-TE) (Figure 1A). 

Live assay. NB-TE samples were incubated in RPMI expansion medium containing 2 μM calcein AM for 30 min at 37 °C, 5% CO_2_, as indicated by the manufacturer’s protocol (LIVE/DEAD^®^ Viability/Cytotoxicity Kit, Molecular Probes, Eugene, OR, USA). The nuclei of the cells were stained with 16.2 mM Hoescht 33342 (Life Technologies, Carlsbad, CA, USA) in PBS. Samples were imaged with a fluorescence microscope (Olympus IX81 light microscope, Center Valley, PA, USA).

Nanoparticle treatment. NB-TE models on day 7 were incubated in exosome-free culture medium containing 50 μg Fe/mL of nanoparticles overnight (O/N) (day 8 after scaffold cell seeding) or for 5 days (day 12 after scaffold cell seeding). Then, supernatants were collected for subsequent EV isolation (Figure 1B).

Internalization Rate Quantification. ImageJ software was employed for quantification. Binary masks were created for each fluorescence channel, and the percentage of area occupied was measured. The internalization rate was determined by dividing the percentage of the area occupied by nanoparticles (red) by the percentage of the area occupied by nuclei (blue). The Y-axis in the graphs represents the internalization rate as a percentage.

Extracellular vesicle isolation and characterization. NB-TE supernatants from controls or nanoparticle-treated samples were collected, and extracellular vesicles were isolated from culture media using the total EV isolation kit (Invitrogen, Waltham, MA, USA), according to the manufacturer’s protocol (Figure 1A,B).

Isolated EVs were resuspended in PBS, and the ζ-potential of the EV control or nanoparticle-containing EVs (Fe-EVs) collected at different time points were determined using a Zetasizer nano series (Malvern, Germany) and a DTS1070 cell. Isolated EVs from a pool of the supernatant of three TE-NB models were assayed for ζ-potential analysis, performing three measurements for each sample and five runs for each measurement.

For particle size distribution and concentration, nanoparticle tracking analysis (NTA) was performed (NanoSight NS300) at the CIBER-BBN/Nanbiosis U6 Biomaterial Processing and Nanostructuring Unit.

The total yield of extracellular EVs was determined through a standardized process. Each TE-NB model, cultured in 3 mL of medium, underwent EV isolation, with resulting EVs resuspended in PBS. NTA was then employed to quantify particle concentrations (particles/mL). Considering the initial culture volume, the total particle count per TE-NB was calculated, presenting this metric as “Total EV yield per 3D model”.

A JEOL 1010 Transmission Electron Microscope (TEM) was used to evaluate extracellular vesicles’ morphology and Fe-NP content. Samples were prepared following a negative staining protocol. Briefly, a droplet of EV suspension was placed on a negatively charged surface, with subsequent incubation under a copper grid coated with formvar for 25 min. After removing the excess sample, the grid was exposed to a 2% uranyl acetate staining solution for 30 s. The excess staining agent was removed, and the grid was air-dried for 2 h.

### 2.2. Histological Studies

TE-NB constructs were washed in PBS and fixed in PFA 37% (Formalin solution 10% neutral buffer, Sigma-Aldrich, St. Louis, MO, USA) overnight at 4 degrees. Then, the samples were processed for histological analysis; they were dehydrated in graded ethanol washes and embedded in paraffin. Serial sections (5 μm thick) were mounted on glass slides and stained using routine hematoxylin/eosin procedures.

To detect Fe-NPs inside the cells, serial sections were stained with Prussian blue (Hematognost Fe staining kit, Sigma-Aldrich, Burghausen, Germany) following the manufacturer’s protocol. Fe-NPs were visualized as free ionic iron stained in blue. Samples were counterstained with the nuclear fast red solution and mounted with a permanent mounting medium (VectaMount, Newark, CA, USA).

## 3. Results

**Figure 2:**
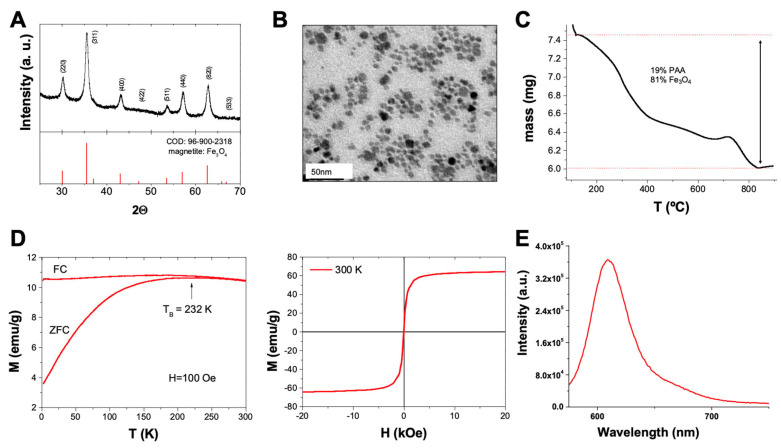
Characterization of polyacrylic acid (PAA)-functionalized magnetite nanoparticles. (**A**) XRD of the polyacrylic acid-coated magnetite nanoparticles showing the COD 96-900-2318 pattern of pure magnetite as reference. (**B**) TEM micrograph of Fe_3_O_4_@PAA nanoparticles functionalized with rhodamine. (**C**) Thermogravimetric analysis of the Fe_3_O_4_@PAA nanoparticles. (**D**) Magnetic properties of the Fe_3_O_4_@PAA nanoparticles: left, hysteresis curve at room temperature between −20 and 20 kOe; right, field-cooled–zero-field-cooled plot at 100 Oe. (**E**) Fluorescence spectra of Fe_3_O_4_@PAA NPs functionalized with rhodamine (excitation wavelength (λex) = 450 nm, slits = 5 nm).

A hydrothermal methodology was used to prepare the functionalized magnetite nanoparticles of polyacrylic acid (PAA). The high quality of these NPs was confirmed by XRD measurements. The diffraction pattern, represented as peaks on the graph, corresponds to specific crystallographic planes in the material. The position, intensity, and shape of these peaks were analyzed to determine the crystal structure, phase composition, and crystallite size of the sample. By XRD, we confirmed (i) the high crystallinity of the sample and (ii) the high purity of the nanoparticles as all peaks observed in the XRD spectrum matched the pattern of magnetite (Fe_3_O_4_; COD 96-900-2318), without extra peaks from other phases (Figure 2A). We used HR-TEM to obtain detailed images of the structure and size of the nanomaterials. We confirmed the pseudo-spherical shape of the nanoparticles with a narrow size distribution and an average size of 9.2 ± 2.5 nm (Figure 2B). TGA was employed to examine the sample, revealing the presence of an organic component originating from the PAA coating (Figure 2C). The TGA results indicated that this organic component constituted approximately 19% of the total sample mass. This information highlights TGA’s capability to discern and quantify different components within the analyzed material based on their thermal behaviors. This organic layer is key to our initial design as it provides the reactive groups (carboxylic groups) for the further functionalization of the samples. Magnetic measurements from the PAA-coated NPs confirmed the superparamagnetic nature of the samples (quasi-zero coercitivity at 300 K and 0 Oe and fast magnetization as the external field increases; and blocking temperature, *T_B_*, below room temperature), which is key to the intended biomedical use of the nanoparticles (Figure 2D). Once the characterization confirmed the adequate quality of the nanoparticles, a sequential functionalization was performed, looking for the follow-up of the internalization and accumulation process in cell culture. Thus, the nanoparticles were modified with a fluorophore (sulforhodamine 101) to provide a simple way to identify/follow them in culture. A cadaverine-modified rhodamine dye was coupled to the NPs using mild peptidic chemistry (EDC/NHS). The fluorescent nature of the Fe_3_O_4_@PAA-Rh was confirmed after purification via fluorescence measurement (Figure 2E).

**Figure 3:**
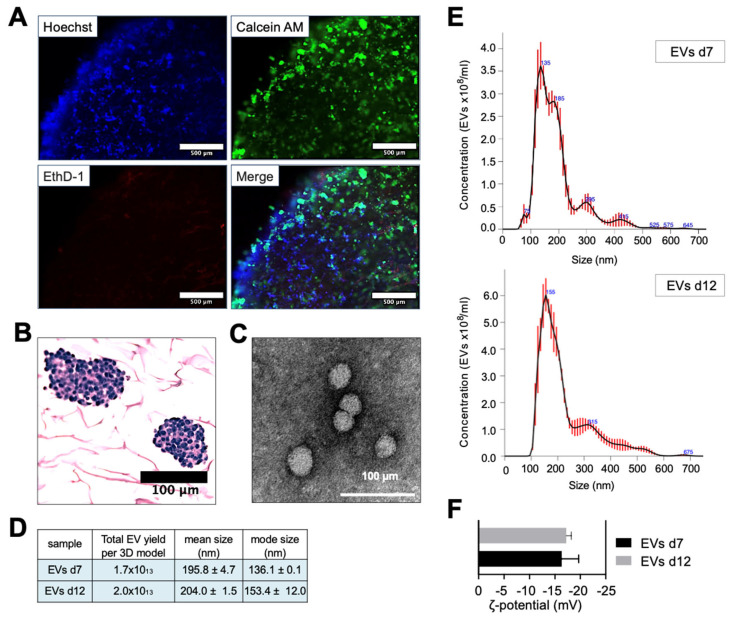
Characterization of extracellular vesicles from long-term tissue-engineered neuroblastoma models. (**A**) Viability analysis of SK-N-BE(2) neuroblastoma cells in tissue-engineered models (TE-NB) on day 12, post-seeding, using live/dead staining. The image depicts a quarter of the TE-NB, showing the edge of the construct and the inner core. Live cells are green (calcein stained), and dead cells are red (ethidium homodimer-1 stained). Representative images are shown (*n* = 12). (**B**) Hematoxylin/eosin staining of TE-NB on day 12 showing SK-N-BE(2) cell aggregates (*n* = 6). (**C**) Transmission electron microscopy (TEM) images of EVs derived from SK-N-BE(2) TE-NB, showing spherical EVs. (**D**) Quantification of total EV yield per 3D model, mean size, and mode size released by SK-N-BE(2) cells in TE-NB on day 12, measured using nanoparticle tracking analysis (NTA). (**E**) Representative size distribution of EVs isolated from TE-NB on day 12, as determined by NTA. (**F**) Zeta potential measurements of EVs isolated at indicated time points.

In previous studies, we demonstrated the stability of ColI/HA-based biomaterials for at least 7 days. We also demonstrated that this time is required for the cells to adapt to the biomimetic environment and start recapitulating native tumor features [17,18]. To establish a protocol for isolating EV-cloaked nanoparticles, we initially determined whether the tissue-engineered models (TE-models) could maintain their viability properties for over a week. Live/dead and histological analyses confirmed the viability and proliferation of neuroblastoma (NB) cells for at least 12 days in vitro (Figure 3A,B). Moreover, EVs isolated from TE-models on day 12 exhibited no discernible differences in morphology (Figure 3C), concentration, size (Figure 3D,E), or zeta potential (Figure 3F) when compared to those isolated on day 7.

The cellular uptake of nanoparticles plays a crucial role in effectively encapsulating these particles within EVs. Typically, the assessment of nanoparticle internalization is conducted within a 2D cell culture model, yielding promising outcomes. Notably, certain nanoparticles, such as IONs, are recognized as efficient carrier systems for internalization in monolayer cultures [19]. Nevertheless, the investigation of nanoparticle internalization in a 3D context remains an area of limited exploration, resulting in a scarcity of available data in this regard.

**Figure 4:**
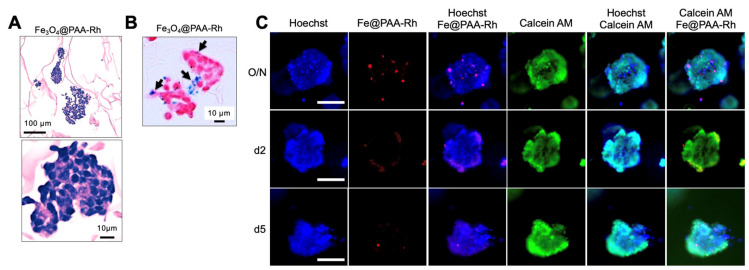
Evaluation of Fe_3_O_4_@PAA-Rh nanoparticle internalization in a 3D neuroblastoma tissue-engineered model (TE-NB). (**A**) Histological analysis of TE-NB after overnight exposure of Fe_3_O_4_@PAA-Rh nanoparticles. Nuclei are stained blue–purple (hematoxylin), and the cytoplasm/extracellular matrix is stained varying shades of pink (eosin). (**B**) Prussian blue staining demonstrating the uptake of iron nanoparticles Fe_3_O_4_@PAA-Rh within TE-NB. Blue staining corresponds to the presence of iron nanoparticles. Arrows indicate nanoparticle accumulation within cells. Nuclei were stained with nuclear fast red solution in pink/red. Representative images are shown (*n* = 6). (**C**) Fluorescence microscopy images capturing the internalization of nanoparticles at various time points (overnight, 2 days, and 5 days) within TE-NB. Cell nuclei were stained by Hoechst 33342, Fe_3_O_4_@PAA-Rh nanoparticles are red, and calcein (live cells) are green. Scale bar = 50 μm. Representative images of three independent assays.

In our study, we sought to investigate the feasibility of labeling NB cells with Fe_3_O_4_@PAA-Rh nanoparticles using a 3-dimensional neuroblastoma TE-model. To achieve this, on day 7, we exposed the TE-model to Fe_3_O_4_@PAA-Rh nanoparticles at a concentration of 50 Fe μg/mL overnight. We employed various methods to assess the presence of nanoparticles within NB cells in the 3D context, including histological analysis (Figure 4A), Prussian blue staining (Figure 4B), and fluorescence microscopy at different time points (overnight, 2 days, and 5 days) (Figure 4C). Interestingly, although the overnight treatment resulted in the highest accumulation of nanoparticles within NB cells, the internalization rate was notably low. Importantly, cell viability, as indicated by calcein AM staining (Figure 4C), remained high after nanoparticle treatment.

**Figure 5:**
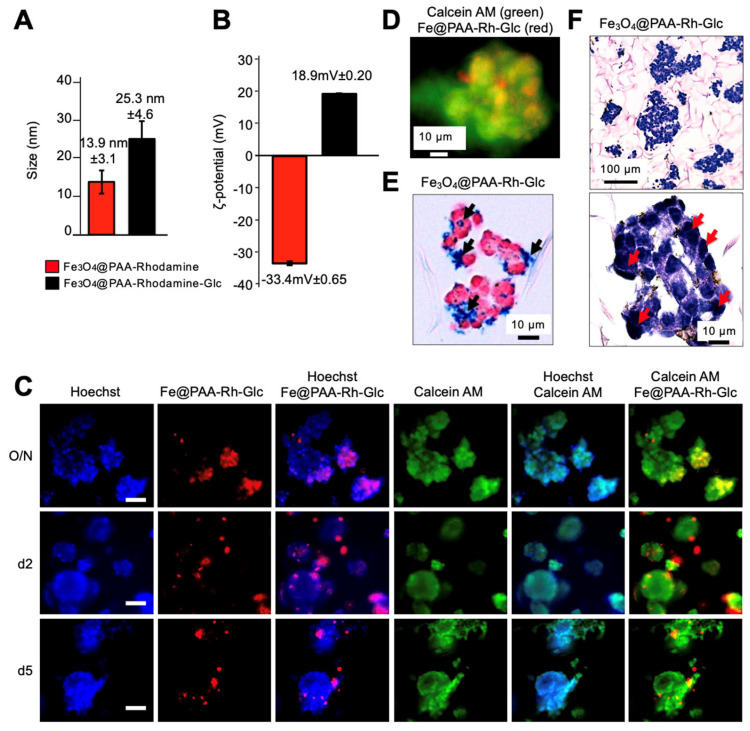
Enhanced internalization of glucose-functionalized Fe_3_O_4_@PAA-Rh nanoparticles in a 3D neuroblastoma tissue-engineered model (TE-NB). (**A**) Hydrodynamic size measurements and (**B**) ζ-potential values of the indicated nanoparticles confirming the successful coupling of glucose to Fe_3_O_4_@PAA-Rh nanoparticles. (**C**) Fluorescence microscopy images depicting the internalization of glucose-modified nanoparticles (Fe_3_O_4_@PAA-Rh-Glc; red) at various time points (overnight, 2 days, and 5 days) within TE-NB. Cell nuclei were stained by Hoechst 33342, and live cells in green by calcein AM. Scale bar = 50 μm. Representative images of three independent assays. (**D**) Fluorescence microscopy image depicting the internalization of glucose-modified nanoparticles after an overnight incubation, captured at higher magnification. Cellular cytoplasm is stained in green using calcein AM, while the nanoparticles are visualized in red. (**E**) Prussian blue staining showing the internalization of Fe_3_O_4_@PAA-Rh-Glc nanoparticles (*n* = 6) and (**F**) histological analysis (hematoxylin/eosin staining).

Cancer cells, including neuroblastoma cells, often exhibit increased glucose metabolism, accompanied by the overexpression of glucose transporters such as GLUT1 [20,21,22]. Leveraging this metabolic characteristic, we aimed to enhance nanoparticle internalization in the 3D context by functionalizing the Fe_3_O_4_@PAA-Rh nanoparticles with glucose. A similar approach to that used for coupling the fluorescent dye was followed for functionalization. Glucosamine served as the starting material, providing the necessary amine groups for coupling to the carboxylic groups of the PAA coating. Both hydrodynamic size measurements and ζ-potential values (Figure 5A,B) confirmed the successful coupling of glucose.

We then evaluated the internalization of these glucose-modified nanoparticles (Fe_3_O_4_@PAA-Rh-Glc) at various time points (overnight, 2 days, and 5 days) using fluorescence microscopy (Figure 5C,D) and overnight through Prussian blue staining (Figure 5E) and histological analysis (Figure 5F). These assessments confirmed a higher degree of internalization of the nanoparticles in the NB cells within the 3D TE-model, compared to what was observed with the Fe_3_O_4_@PAA-Rh nanoparticles (Figure 4A–C). Importantly, no toxic effects were detected, as evidenced by the calcein AM staining results (Figure 5C).

**Figure 6:**
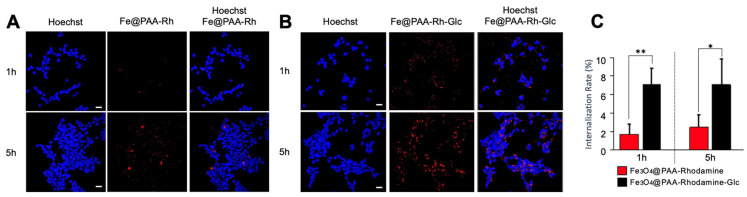
Evaluation of Fe_3_O_4_@PAA-Rh and Fe_3_O_4_@PAA-Rh-Glc nanoparticle internalization in two-dimensional SK-N-BE(2) cell cultures. (**A**) Confocal microscopy images illustrating the internalization of Fe_3_O_4_@PAA-Rh nanoparticles (red) at various time points (1 h and 5 h) within 2D cell culture. Cell nuclei were stained with Hoechst 33342. Scale bar = 25 μm. (**B**) Confocal microscopy images depicting the internalization of glucose-modified nanoparticles (Fe_3_O_4_@PAA-Rh-Glc; red) at various time points (1 h and 5 h) within 2D cell culture. Cell nuclei were stained with Hoechst 33342. Scale bar = 25 μm. (**C**) Quantification of internalization rate in two-dimensional cultures, calculated using ImageJ. The Y-axis represents the internalization rate (%), calculated as the percentage of area occupied by nanoparticles (red) divided by the percentage of area occupied by nuclei (blue) (*n* = 3 per condition). Error bars represent standard deviations. Statistical significance was determined by the two-tailed Student’s *t*-test. ** *p* < 0.01; * *p* < 0.05.

Taken together, all these studies demonstrated superior cell internalization of glucose-functionalized NPs in 3D. To investigate the impact of the 3D tumor microenvironment on the internalization rate of Fe_3_O_4_@PAA-Rh nanoparticles compared to their glucose-functionalized counterparts, we also conducted experiments on NB cells cultured in 2D settings. Recognizing the rapid internalization facilitated by gravity in 2D cultures, we established a time frame of 1 to 5 h to discern internalization differences in NP conditions. At the 1 h incubation, glucose-functionalized NPs exhibited significantly higher cell internalization than the non-functionalized NPs (Figure 6). This trend persisted up to the 5 h, where we observed a convergence in internalization rates between the two NP conditions (Figure 6). These results indicate that adding glucose to the NPs promotes heightened cell internalization, a consistent finding across both 2D and 3D cell culture models. Consequently, this type of NP was selected for further investigations.

**Figure 7:**
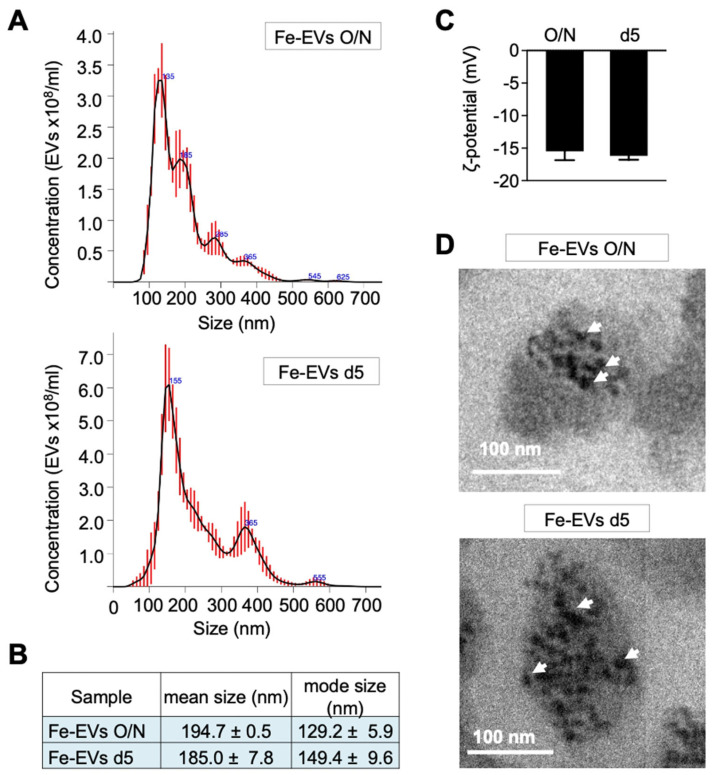
Characterization of glucose-functionalized nanoparticle-loaded extracellular vesicles (Fe-EVs) isolated at different time points. (**A**) Representative size distribution of EVs loaded with Fe@PAA-Rh-Glc nanoparticles (Fe-EVs) and isolated from TE-NB immediately after NP treatment (Fe-EVs O/N; day 8 after SKN-BE(2) cell seeding) and after 4 days of treatment (Fe-EVs d5; day 12 after SKN-BE(2) cell seeding), as determined by nanoparticle tracking analysis (NTA). (**B**) Quantification of Fe-EV concentration, mean size, and mode size released by SK-N-BE(2) cells in TE-NB on the indicated time points (overnight = O/N and day 5 = d5), measured using NTA. (**C**) Zeta potential measurements of Fe-EVs isolated at indicated time points. (**D**) Transmission electron microscopy (TEM) images confirming the presence of Fe-NPs within EVs at indicated time points. The white arrows point to Fe-NPs inside the EV. Representative images of three independent assays.

Finally, TE-models on day 7 were treated overnight with glucose-functionalized NPs, and EVs were isolated at two different time points: immediately after the treatment (Fe-EVs O/N) and after 4 days of treatment (Fe-EVs d5) (Figure 1B). No significant differences were observed in terms of size (Figure 7A,B). To investigate the encapsulation of Fe-NPs within the EVs, we employed zeta potential analysis, a technique crucial for understanding the surface charge characteristics of nanoparticles and their interactions with EVs. The zeta potential of EVs alone was consistently negative at approximately −15 mV (Figure 3F), while the Fe-NPs functionalized with glucose exhibited a positive zeta potential of +18.9 mV +/− 0.20 (Figure 5B). Importantly, when loaded with Fe-NPs, the zeta potential of the EVs remained negative and consistently around −15 mV (Figure 7C). This observation suggested that the Fe@PAA-Rh-Glc nanoparticles were likely encapsulated within the EVs. The absence of a deviation from the typical negative zeta potential of EVs, which would be expected if the nanoparticles were merely bound to the EV surface, reinforced the conclusion that the loaded nanoparticles are situated inside the EVs. TEM analysis was conducted to provide additional confirmation, confirming the presence of iron NPs within the EV structures at both the overnight and day-5 time points (Figure 7D). These findings collectively support that the glucose-functionalized Fe NPs are indeed encapsulated within the EVs and not merely bound to the EV surface.

## 4. Discussion

In this research, we delved into incorporating iron oxide nanoparticles into EV-derived neuroblastoma cells using a method called parental labeling but with the challenge of using 3D-engineered niches.

The parental labeling method stands out as a cutting-edge approach for encapsulating NPs within EVs, offering notable advantages in terms of biological relevance, efficiency, and versatility. Comparing the parental labeling method with other conventional techniques, it is essential to highlight some key distinctions and advantages. While many methods rely on post-isolation manipulation of EVs or physically forcing NPs into pre-existing vesicles, parental labeling leverages the natural cellular processes of NP internalization and EV biogenesis. This method closely mirrors physiological processes, ensuring minimal perturbation to cellular physiology and maintaining biological relevance. However, its application within 3D models or biologically relevant systems presents a unique set of challenges.

Compared to conventional 2D cultures, adopting 3D models introduces complexity and optimization hurdles due to the altered diffusion gradients inherent in 3D environments. Achieving efficient distribution and uptake of NPs by parent cells becomes an intricate task. Also, in the 3D landscape, cellular heterogeneity becomes notably pronounced. Different cell populations may exhibit varying degrees of NP uptake. Extended processing times are another challenge associated with parental labeling within 3D systems. Ensuring the efficient internalization of NPs by parent cells and subsequent EV biogenesis often demands longer culture periods. This extended timeline introduces the potential for variability or alterations to the 3D model over time, necessitating careful monitoring and control. Additionally, the biocompatibility and potential toxicity of NPs may manifest differently within 3D models compared to traditional 2D cultures.

We conducted parental labeling within a sophisticated 3-dimensional biomimetic model of neuroblastoma, a particularly deadly type of pediatric cancer. We first evaluated the tissue-engineered model to maintain the viability and proliferation of NB cells for at least 12 days, demonstrating their utility as a platform for EV isolation. Importantly, EVs obtained from TE-models at long-term cultures exhibited no discernible differences in morphology, concentration, size, or zeta potential compared to those isolated at short-term cultures. Moreover, we observed high cell viability after NP treatment, as indicated by calcein AM staining (Figure 4C and Figure 5C). This signifies that the nanoparticles used in our 3D model are biocompatible and do not negatively affect cell health. This outcome supports the feasibility of our parental labeling method for nanoparticle encapsulation within 3D systems while preserving cell viability.

The cellular uptake of nanoparticles is crucial in their effective encapsulation within EVs. The extent of nanoparticle internalization by host cells directly and profoundly impacts the quantity of nanoparticles encapsulated within EVs. Low internalization results in fewer nanoparticles available for inclusion, leading to a lower cargo composition of nanoparticles within the EVs. This highlights the importance of strategies to optimize internalization for maximizing the yield and effectiveness of EVs loaded with nanoparticles in various biomedical applications.

Also, the selection of EV isolation methods necessitates a meticulous evaluation of their inherent advantages and limitations. While a magnet-based isolation strategy offers specific advantages, particularly for targeted isolation of EV-loaded IONs, direct isolation from tissue-engineered models raises concerns about potential membrane fragment capture, which may impact EV integrity and model longevity. Similarly, indirect isolation from the cell culture using a magnet poses risks of EV membrane disruption and the collection of free nanoparticles. In our study, we opted for a commercially available isolation kit designed for total EV isolation, aiming to provide a comprehensive representation of EV subpopulations. The advantage of using isolation kits lies in their simplicity, time efficiency, and capacity to capture a diverse range of EVs. However, it is essential to acknowledge potential drawbacks, including challenges in achieving absolute purity and the potential co-isolation of non-NP-loaded EV entities. The decision to employ an isolation kit aligned with our goal of striking a balance between loading efficiency and preserving the biomimetic properties of EVs, particularly their intact state with unique surface ligands for precise cellular uptake and targeting specificity. Moving forward, efforts will concentrate on refining loading quantification metrics and exploring alternative methodologies that optimize both efficiency and scalability. The dynamic landscape of isolation techniques and loading strategies presents an exciting avenue for ongoing research and optimization, promising enhanced precision in NP-loaded EV delivery systems.

While previous studies have predominantly focused on 2D cell culture models, our study investigated nanoparticle internalization within a 3D context. We observed that overnight treatment resulted in the highest accumulation of nanoparticles within NB cells in the 3D TE-model. However, the internalization rate of Fe_3_O_4_@PAA-Rh was relatively low, highlighting the need for further optimization. Within this 3D niche, various factors, such as cellular architecture, extracellular matrix interactions, and diffusion, could play pivotal roles in modulating nanoparticle uptake. To address this challenge and enhance the efficacy of nanoparticle internalization, we explored a novel approach to glucose functionalization. Cancer cells, including neuroblastoma cells, are known for their heightened glucose metabolism and overexpression of glucose transporters such as GLUT1 [20,21,22]. Leveraging this metabolic characteristic, we functionalized the iron oxide nanoparticles with glucose moieties in an effort to facilitate their uptake by cancer cells.

Results from the 2D experiments, conducted at 1 h and 5 h incubation periods, revealed a significantly higher internalization rate for glucose-functionalized NPs compared to their non-functionalized counterparts. These findings underscore the effectiveness of glucose functionalization in promoting rapid and enhanced internalization of NPs, even in the comparatively swift internalization environment of 2D cultures. The positively charged glucose-modified NPs demonstrated superior uptake, likely through interactions with cellular membranes, showcasing the potential of this functionalization strategy for improving NP cell internalization. These insights from 2D experiments strengthen the rationale for pursuing glucose functionalization as a means to enhance NP uptake. Furthermore, as determined by zeta potential studies, the positive charge observed on glucose-modified nanoparticles could also play a pivotal role in strengthening their cellular internalization within the intricate 3D tumor microenvironment. This positive charge serves a multifaceted purpose in facilitating NP uptake because it engenders electrostatic attraction, enabling positively charged NPs to establish favorable interactions with negatively charged cell membranes. This attraction fosters initial contact and adhesion of NPs to the cell surface, thereby heightening the prospects of internalization. Also, the positive charge on NPs could augment their cellular uptake by facilitating entry through endocytic pathways, including clathrin-mediated or caveolin-mediated endocytosis. This internalization mechanism is particularly potent when NPs bear a net positive charge, further accentuating their capacity to be taken up by cells. Together, our findings demonstrated a remarkable improvement in nanoparticle internalization in 2D and within the 3D neuroblastoma TE-model upon introducing glucose-functionalized nanoparticles. This enhancement suggests that glucose modification not only capitalizes on the metabolic preferences of cancer cells but also potentially interacts with overexpressed GLUT1 transporters, facilitating more efficient cellular uptake [20,21,22]. This possible dual mechanism, involving the positive charge on NPs and the specific targeting of GLUT1 transporters, synergistically could contribute to the enhanced cell internalization observed in our study. This selective affinity enables these NPs to target and potentially improve their uptake by cancer cells within the tumor microenvironment.

Biomimetic 3D models closely emulate the native tissue architecture, cell–cell interactions, and extracellular matrix composition, thus providing a more accurate representation of the physiological environment. This precision is particularly crucial when it comes to NP-loaded EVs, as it enables the assessment of their behavior in a context that mirrors the in vivo scenario more closely [23]. Moreover, EVs isolated from biomimetic 3D models inherit the surface ligands and targeting properties that reflect the specific tumor microenvironment. This means that they carry the inherent ability to selectively bind to cancer cell receptors, thus enhancing the specificity of NP delivery. In contrast, traditional 2D monolayer cultures lack these complexities and targeting capabilities, making them less effective in distinguishing between cancer and healthy cells. As a result, using EVs derived from 3D models is starting to be a promising strategy to significantly improve the precision of NP delivery to cancer cells while reducing the risk of nonspecific uptake by non-cancerous cells, thereby increasing the therapeutic potential and minimizing potential side effects [24,25].

In conclusion, in this study, we have introduced several innovative approaches that significantly advance the field of nanoparticle delivery.

Firstly, the parental labeling method has emerged as a versatile and biologically relevant approach for encapsulating NPs within EVs. By closely mirroring physiological processes, this method offers minimal disruption to cellular physiology and maintains biological relevance. However, its application within 3D models presents challenges, including complexity, cellular heterogeneity, extended processing times, and considerations of biocompatibility and potential toxicity. Our experiments with glucose-modified nanoparticles to enhance internalization within a 3D biomimetic neuroblastoma model overcome the mentioned issues and demonstrate the approach’s viability. Additionally, the positive charge observed on glucose-modified NPs seems to also play a pivotal role in bolstering their cellular internalization within the 3D tumor microenvironment. Importantly, we observed high cell viability after NP treatment, indicating the biocompatibility of NPs within the 3D system. This result supports the feasibility of parental labeling for NP encapsulation within 3D systems while preserving cell health.

Secondly, the incorporation of 3D bioengineered models for the generation of EVs used as carriers for nanoparticles adds a critical dimension to our methodology. Utilizing EVs derived from TE-models as carriers provides a more advanced and cancer-specific approach [24,25]. These EVs closely mimic the surface ligands and targeting properties of their in vivo counterparts, making them ideal for achieving precise and effective tumor targeting.

Lastly, encapsulating nanoparticles within EVs offers a distinct advantage. It shields the nanoparticles from potential immune responses and degradation and enhances their stability, ensuring precise targeting and integrity during transit to the target cells. These innovations pave the way for more efficient and cancer-specific drug delivery systems, bringing us closer to personalized and effective cancer therapies.

## Data Availability

The datasets used during and/or analyzed during the current study are available from the corresponding author upon reasonable request.
